# Tao brush endometrial cytology is a sensitive diagnostic tool for cancer and hyperplasia among women presenting to clinic with abnormal uterine bleeding

**DOI:** 10.1002/cam4.4235

**Published:** 2021-09-16

**Authors:** Stephanie R. DeJong, Jamie N. Bakkum‐Gamez, Amy C. Clayton, Michael R. Henry, Gary L. Keeney, Jun Zhang, Trynda N. Kroneman, Shannon K. Laughlin‐Tommaso, Lisa J. Ahlberg, Ann L. VanOosten, Amy L. Weaver, Nicolas Wentzensen, Sarah E. Kerr

**Affiliations:** ^1^ Department of Obstetrics and Gynecology Division of Gynecologic Surgery Mayo Clinic Rochester MN USA; ^2^ Department of Anatomic Pathology Mayo Clinic Rochester MN USA; ^3^ Department of Anatomic Pathology Mayo Clinic Phoenix AZ USA; ^4^ Department of Obstetrics and Gynecology Division of Gynecology Mayo Clinic Rochester MN USA; ^5^ Department of Obstetrics and Gynecology Division of Obstetrics and Gynecology Research Mayo Clinic Rochester MN USA; ^6^ Department of Health Sciences Research Mayo Clinic Rochester MN USA; ^7^ Division of Cancer Epidemiology and Genetics National Cancer Institute Bethesda MD USA; ^8^ Currently: Hospital Pathology Associates Minneapolis MN USA

**Keywords:** endometrial biopsy, endometrial cancer, endometrial cytology, endometrial hyperplasia, Tao brush

## Abstract

**Background:**

Abnormal uterine bleeding requires the investigation of the endometrium. Histology is typically used but there remains room for the improvement and use of cytology.

**Methods:**

Women presenting for clinically indicated office endometrial biopsy were prospectively enrolled. Tao endometrial brushing and office endometrial biopsy were performed, and surgical procedure if clinically indicated. Tao brush cytology specimens were blindly reviewed by up to three pathologists, consensus obtained, and scored as: benign, atypical (favor benign), suspicious, positive for malignancy, or non‐diagnostic. Cytology and histology were compared to surgical pathology to determine sensitivity, specificity, positive, and negative predictive values to detect AH (atypical hyperplasia) or EC (endometrial cancer).

**Results:**

Clinical indications of 197 enrolled patients included postmenopausal bleeding (90, 45.7%), abnormal uterine bleeding (94, 47.7%), and abnormal endometrium on ultrasound without bleeding (13, 6.6%). Of the 197 patients, 185 (93.9%) had cytology score consensus and a total of 196 (99.5%) had consensus regarding cytology positivity.

Surgical pathology diagnoses (N = 85) were 13 (15.3%) FIGO grade 1 or 2 EC, 3 (3.5%) AH, and 69 (81.2%) benign endometrium. Sensitivity and specificity to detect EC or AH were 93.7% and 100%, respectively, via endometrial biopsy; 87.5% and 63.8%, respectively, via endometrial cytology when scores of malignancy, suspicious, or atypical were considered positive.

**Conclusions:**

In a high‐risk population, Tao brush endometrial cytology showed high sensitivity to detect AH and EC comparable to biopsy histology when considering scores of malignancy, suspicious, atypical, and non‐diagnostic. Revisiting the potential value of endometrial cytology in the contemporary era of endometrial diagnostic workup is warranted.

## Graphical abstract

Tao brush cytology is a sensitive tool for endometrial cancer evaluation in symptomatic women. There is a high concordance of Tao brush readings amongst blinded pathologists

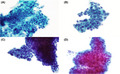

## 1 INTRODUCTION

Abnormal uterine bleeding (AUB) is the leading indication for referral to a gynecologist^1^ and is used to describe menstrual flow outside of normal parameters.^2^ Often the cause of AUB is benign but in a fraction of women the bleeding signals something more serious such as endometrial cancer (EC).^3^ Postmenopausal bleeding (PMB) is now defined separately from AUB as any uterine bleeding occurring after 1 year of amenorrhea^4^ and incidence of malignancy is up to 14% in this group.^5^, ^6^ The American College of Obstetricians and Gynecologists (ACOG) recommends ultrasound and endometrial sampling for women with EC risk factors and AUB or PMB.^5^, ^7^ This can be completed in the office as an endometrial biopsy using an aspiration method or in the operating room as a dilation and curettage (D&C) or directed biopsy or endometrial lesion resection with hysteroscopic guidance.^8^ Unfortunately, studies have demonstrated that even aggressive D&C in the operating room evaluates less than 50% of the uterine cavity in the majority of procedures.^9^ Pipelle has been reported to sample 4% of the endometrial surface and concordance with pathology diagnosis on hysterectomy is 83.8%^10^

The Tao brush was approved by the United States Food and Drug Administration (FDA) in 1993 as a device for endometrial sampling and cytological examination.^11^ It consists of a sheathed, cylindrical 3mm brush that is inserted trans‐cervically into the uterine cavity for direct epithelial brushing. Supporting data for FDA approval illustrated that the device was 95.5%–100% effective in detecting endometrial cancer when compared with the Pipelle endometrial biopsy device.^12^ The technique for Tao brush sampling is simple to learn, and the device appears to sample more endometrial surface area compared to traditional biopsy methods or even the criterion standard for endometrial sampling which is D&C.^13^ The sheathed brush design also allows for the sampling of the endometrium while avoiding endocervical contamination^14^ and decreasing patient discomfort.^15^, ^16^ It has been suggested that endometrial samples are more adequately and consistently obtained even from nulliparous and postmenopausal women compared to traditional office endometrial biopsy devices.^13^ Additionally, comparable diagnostic sensitivity between Tao brush and endometrial biopsy for EC and atypical hyperplasia (AH) has been demonstrated in studies performed on hysterectomy specimens.^12^, ^17^ The accuracy of Tao brush cytology has been reported to be between 88.9% for EC and 85.7% for complex atypical hyperplasia (CAH) when cytology is performed in the operating room at the time of D&C^17^ and up to 96% concordance with surgical pathology in a large review.^18^ However, data have been discordant in demonstrating the sensitivity and specificity for the detection of benign pathology.^12^, ^16^, ^19^, ^20^ Some reports demonstrate 86.7% sensitivity in detecting endometritis and 77.8% sensitivity in detecting endometrial polyps, neither of which are significantly different from D&C.^16^

Although the Tao brush was approved by the FDA in 1993, its application has been limited. Diagnosis of endometrial pathology by this sampling method has traditionally required a pathologist that specializes in cytology^21^, ^22^ and even cytopathologists are not routinely trained in endometrial cytology interpretation. However, as advances have been made in uterine cervical cytology including liquid‐based preparation and automated workflows^23^, ^24^; revisiting the potential of cytology for endometrial pathology diagnosis and EC screening is warranted. As has already occurred with current approaches to Pap test cytology, a standardized, automated endometrial cytology interpretation could lead to major advancement in the field of AUB and postmenopausal bleeding (PMB) diagnostics.

At present, direct comparison performance data between endometrial cytology and endometrial biopsy are lacking. Although early data suggest that endometrial cytology as a diagnostic for AH and EC has acceptable performance,^12^, ^16^ data from recent cohorts with the review of cytology specimens by general pathologists are not available. Additionally, studies comparing biopsies obtained in the clinic setting from symptomatic women both with office endometrial brushing cytology and office biopsy histology against surgical specimens as the criterion standard is needed. The aim of this study is to compare the diagnostic accuracy of Tao brush cytology and office endometrial biopsy in identifying AH and EC among women presenting for endometrial biopsy indications of AUB, PMB, or abnormal endometrial ultrasound findings without bleeding and who had a subsequent surgical diagnostic or therapeutic procedure.

## 2 METHODS

### 2.1 Patient selection

Women presenting to the Abnormal Uterine Bleeding Clinic for a clinically indicated office endometrial biopsy were prospectively enrolled between February 2013 and August 2015, in the feasibility period of a large ongoing prospective cohort study.^25^, ^26^ Women were eligible if they were ≥45 years of age with AUB, PMB, or abnormal ultrasound without bleeding. Abnormal ultrasound was defined as thickened endometrial stripe (>4 mm in asymptomatic postmenopausal women^27^) or endometrial mass identified on ultrasound. Exclusion criteria included prior pelvic radiation, cervical stenosis precluding endometrial sampling, or Lynch Syndrome. Patients were further excluded if they had an inadequate office endometrial biopsy. Eligibility criteria, demographic information, symptoms, and medical history were recorded. Electronic medical records were also abstracted for follow‐up data such as surgical diagnostic or therapeutic procedures of D&C, operative hysteroscopy, or hysterectomy completed before the end of 2016. Office endometrial biopsy was performed using either Endosampler or Pipelle device and device type was recorded. Tao brush samples and endometrial biopsies were both collected during the same office visit. Institutional Review Board approval was obtained for this study.

**FIGURE 1:**
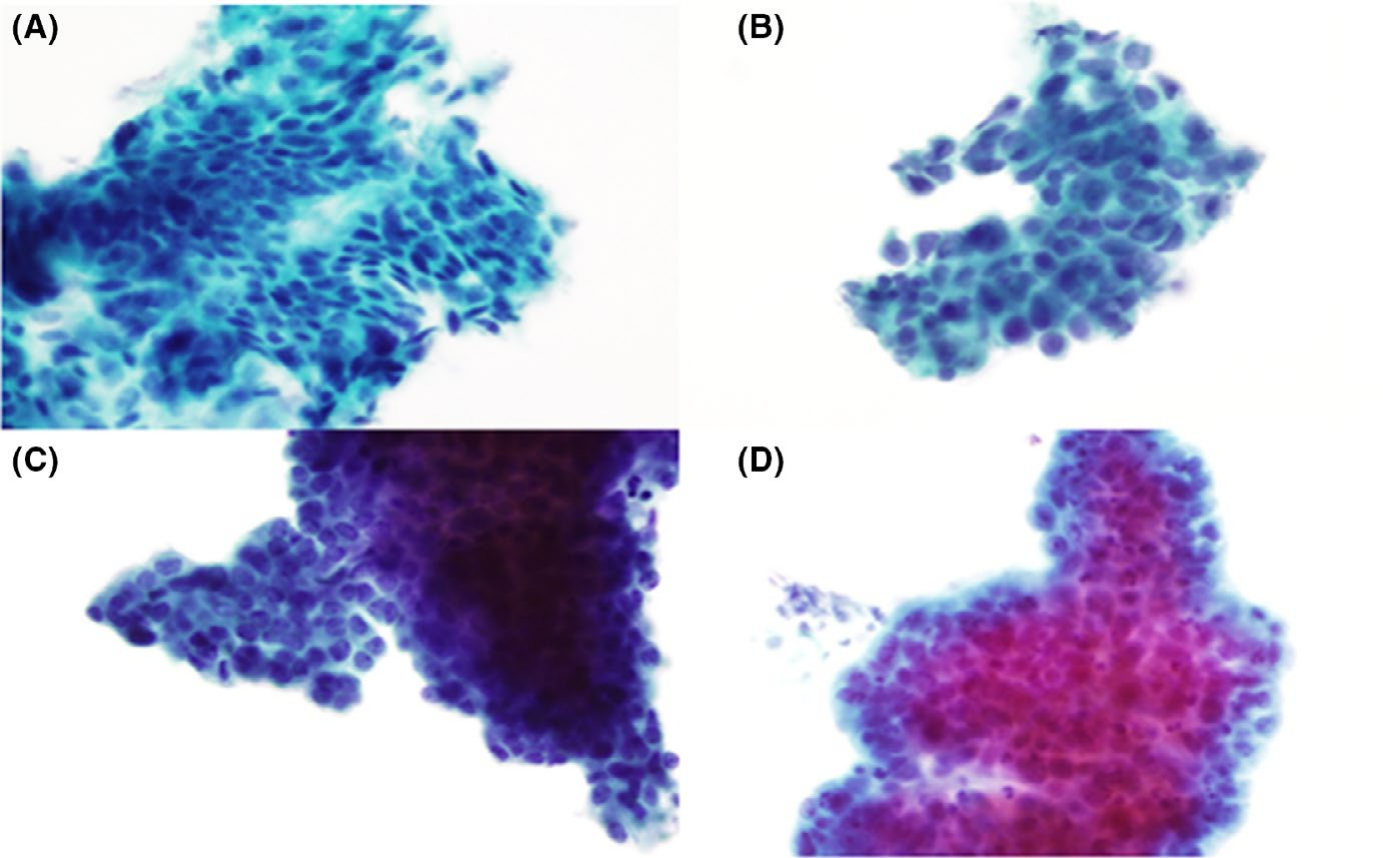
Endometrial cytology cytomorphologic features. Panel (A) benign endometrium. Panel (B) atypical endometrium. Panel (C) suspicious endometrium. Panel (D) malignant endometrium

Specimen collection and processing:

Endometrial tissue obtained via office endometrial biopsy was fixed in formalin. Endometrial biopsy pathology was interpreted per clinical standard of care by pathologists. After Tao endometrial brushing was completed, the bristled tip of the brush was removed from the Tao device using a wire cutter and placed in PreservCyt. In the cytopathology laboratory, the brush was scraped and the resulting material was placed back into the same PreservCyt vial. A monolayer cytology slide was made after glacial acetic acid treatment, using the Thin Prep 2000 Processor (Hologic) and Papanicolaou stained. Each sample was pre‐screened by a cytotechnologist, and then randomly assigned to be blindly reviewed by up to three of five study pathologists (SEK, ACC, MRH, GLK, and JZ). Each sample was scored as: benign, atypical (favor benign), suspicious, positive for malignancy, or non‐diagnostic (did not meet criteria for another score) (Figure 1). All study pathologists underwent a brief pre‐study training with SEK demonstrating photographs and glass slide examples from each scoring category. The training examples were independent of samples from the prospective study. A final cytology score was assigned based on agreement by at least two pathologists. Patients were further excluded if the final cytology score was non‐diagnostic. Surgical pathology from D&C, operative hysteroscopy, or hysterectomy was interpreted by pathologists via clinical standard of care and were the study criterion standard for final surgical pathology diagnoses.

### 2.2 Statistical analysis

Data were summarized using standard descriptive statistics. Endometrial biopsy histology diagnoses of AH, EC, or non‐diagnostic were grouped as positive. Cytology diagnoses from Tao brush sampling were analyzed with two different categorizations: positive for malignancy, suspicious, or atypical were considered positive for criteria A; and positive for malignancy or suspicious were considered positive for criteria B. Diagnostic performances of cytology and office endometrial biopsy were compared to the criterion standard of surgical pathology to determine sensitivity, specificity, positive, and negative predictive values. Ninety‐five percent confidence intervals were constructed using exact methods for a binomial parameter. A two‐sided McNemar's test for comparing correlated proportions was used to compare sensitivity (and likewise specificity) between cytology and endometrial biopsy. Statistical analysis was performed using the SAS version 9.4 software package (SAS Institute, Inc.).

## 3 RESULTS

### 3.1 Baseline characteristics

One hundred and ninety‐seven patients met the study inclusion criteria and had an adequate endometrial biopsy along with Tao Brush sampling. The mean age among the 197 patients was 55.0 (SD 7.6) years with 98 (49.7%) postmenopausal (Table 1). Clinical indications for endometrial biopsy included PMB (90, 45.7%), AUB (94, 47.7%), and abnormal ultrasound without uterine bleeding (13, 6,6%). Among the 197 patients, 188 had the time recorded for both endometrial biopsy and Tao brush collection: 86 (45.7%) patients had office endometrial biopsy collected first, 91 (48.4%) had Tao brushing collected first, and 11 (5.9%) had simultaneous times recorded.

**TABLE 1:** Baseline characteristics of patients presenting to abnormal uterine bleeding clinic at mayo clinic

Characteristic	All patients with endometrial biopsy and Tao brush cytology (N = 197)	Patients with endometrial biopsy, Tao brush cytology, and surgery (N = 85)
Age (years)		
Mean (SD)	55.0 (7.6)	56.8 (8.0)
Range	45.1–78.5	45.3–78.5
Menopausal status, N (%)		
Pre‐ or Peri‐	94 (47.7%)	30 (35.3%)
Post‐	98 (49.7%)	51 (60.0%)
Uncertain	5 (2.5%)	4 (4.7%)
Body mass index (kg/m^2^)		
Mean (SD)	31.7 (8.0)	33.9 (8.6)
Range	18.3–58.6	18.3–58.6
Indication for initial evaluation, N (%)^a^		
PMB	90 (45.7%)	48 (56.5%)
AUB	94 (47.7%)	31 (36.5%)
Abnormal ultrasound without		
Uterine bleeding	13 (6.6%)	6 (7.1%)

Abbreviations: AUB, abnormal uterine bleeding; DUB, dysfunctional uterine bleeding; PMB, postmenopausal bleeding.

a Indication based on the following hierarchy: PMB, AUB or DUB, and abnormal ultrasound without uterine bleeding.

### 3.2 Pathologist agreement of Tao brush sampling

**FIGURE 2:**
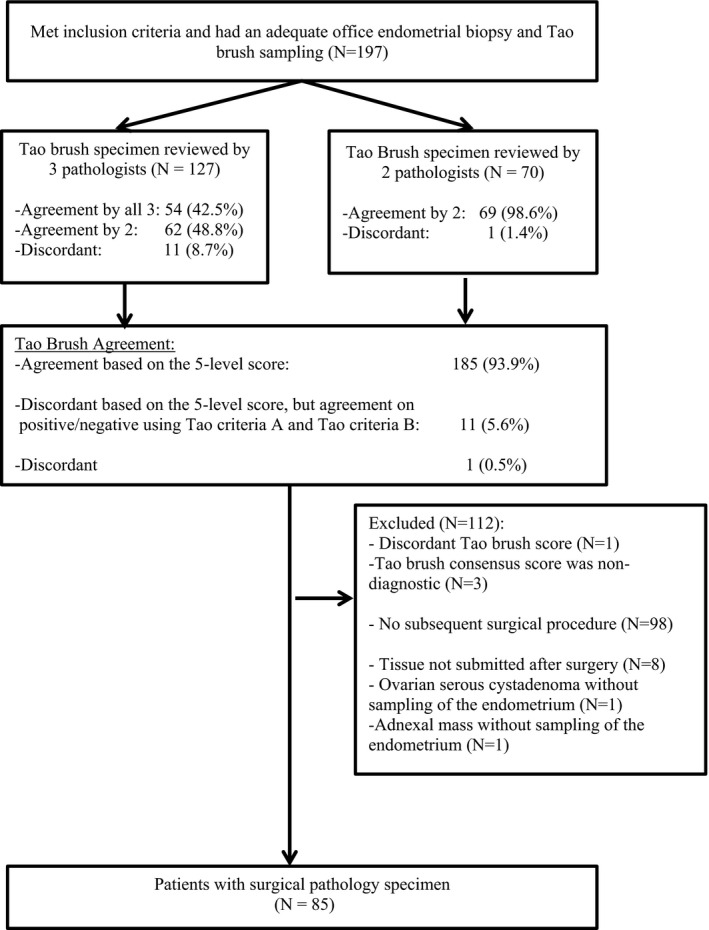
Patient selection and study schema

All endometrial cytology samples were first reviewed by cytotechnologists. Cytology specimens were then reviewed and scored by 3 of the 5 study pathologists at random assignment for 127 patients and by 2 of the 5 study pathologists at random for 70 patients. As outlined in Figure 2, there was exact agreement using the 5‐level scoring by at least 2 of the 3 pathologists for 185 (93.9%) of the 197 patients, including 6 positive for malignancy, 25 suspicious, 32 atypical (favor benign), and 119 negative and 3 non‐diagnostic. Among the remaining 12 patients, there was exact agreement by at least 2 of the 3 pathologists on positive/negative status when applying either criteria A or criteria B for 11 of the 12; all 11 were positive using criteria A and all 11 were negative using criteria B. Therefore, upon excluding the 1 patient with discordant ratings and the 3 scored as non‐diagnostic, 193 had a final Tao Brush cytology score for analysis.

### 3.3 Surgical findings

Ninety‐five of the 193 patients with a Tao brush cytology score underwent a subsequent surgical diagnostic or therapeutic procedure, however, tissue was not sampled for 8 patients and 2 patients had ovarian pathology without a sampling of the endometrium. This yielded 85 patients who had specimens from all three sources (Tao brush pathology, endometrial biopsy, surgical pathology; Figure 2). Their baseline characteristics are summarized in Table 1. Surgical pathology findings following D&C, operative hysteroscopy or hysterectomy were: 7 (8.2%) FIGO grade 1 endometrioid ECs, 6 (7.1%) FIGO grade 2 endometrioid ECs, 3 (3.5%) AH, and 25 (29.4%) benign endometrium (which included simple hyperplasia without atypia (2), fibroids (1), proliferative endometrium (13), disordered (1), secretory (1), atrophic (1), inactive (3) or benign NOS(3)) and 44 polyps (51.8%) (Table 2).

**TABLE 2:** Final pathologic diagnosis from surgical specimen

Final pathology	N (% of 85)
FIGO grade 1 endometrial cancer	7 (8.2%)
FIGO grade 2 endometrial cancer	6 (7.1%)
Atypical hyperplasia	3 (3.5%)
Benign endometrium (including polyps)	69 (81.2%)

Abbreviation: FIGO, Federation of Gynecology and Obstetrics.

### 3.4 Performance of endometrial sampling methods for detecting malignancy

Among the 85 patients with all 3 specimens, sensitivity and specificity of office endometrial biopsy to detect EC or AH were 93.7% and 100%, respectively. For the endometrial cytology from the Tao brush sampling, sensitivity and specificity were 87.5% and 63.8%, when scores of positive for malignancy, suspicious, or atypical were considered positive (criteria A in Table 3). When scores of positive for malignancy, or suspicious were considered positive, sensitivity and specificity for cytology were and 75.0% and 84.1%, respectively (criteria B in Table 3). The sensitivity of the office endometrial biopsy was not significantly different from that of the Tao brush sampling for either criteria A or criteria B (*p* = 0.32 and *p* = 0.08, respectively), but the statistical power was limited with only 16 patients with EC or AH on surgical pathology. The office endometrial biopsy was significantly more specific than Tao brush sampling for criteria A (*p *< 0.001) but not for criteria B (*p* = 0.11).

**TABLE 3:** Performance comparison of office endometrial biopsy and tao brush endometrial cytology based on 85 patients

Performance characteristic	Office endometrial biopsy^a^		Tao brush endometrial cytology, criteria A^b^		Tao brush endometrial cytology, criteria B^c^	
	Rate	95% CI	Rate	95% CI	Rate	95% CI
Sensitivity, %	93.7 (15/16)	69.8–99.8	87.5 (14/16)	61.6–98.4	75.0 (12/16)	47.6–92.7
Specificity, %	100 (69/69)	94.8–100	63.8 (44/69)	51.3–75.0	84.1 (58/69)	73.3–91.8
PPV, %	100 (15/15)	78.2–100	35.9 (14/39)	21.2–52.8	52.2 (12/23)	30.6–73.2
NPV, %	98.6 (69/70)	92.3–100	95.6 (44/46)	85.2–99.5	93.6 (58/62)	84.3–98.2

Abbreviations: CI, confidence interval; NPV, negative predictive value; PPV, positive predictive value.

^a^ A positive office endometrial biopsy included positive for malignancy (n = 10) or atypical hyperplasia (n = 5)

^b^ Criteria A, positive Tao Brush was defined based on cytology findings of positive for malignancy, atypical, or suspicious.

^c^ Criteria B, positive Tao Brush was defined based on cytology findings of positive for malignancy or suspicious.

## 4 DISCUSSION

The high sensitivity (87.5%) of Tao brush cytology in this study supports further evaluation of endometrial sampling by brushing as a useful diagnostic approach for AH and EC in symptomatic women undergoing outpatient clinic evaluation. Our findings are consistent with previously reported data^28^ of the high sensitivity of endometrial cytology to diagnose AH and EC in hysterectomy specimens with known diagnoses.^17^, ^29^ Although tissue sampling is considered the current clinical reference standard, sensitivity was not significantly different between the two diagnostic approaches in this study.

The Tao brush is a device that has been suggested to provide a more complete sampling of the endometrial cavity due to the flexibility and design of the device.^13^, ^29^ Additionally, the monolayer preparation of the collected cells may allow for a more comprehensive assessment of the epithelium.^28^, ^30^ Previous studies have suggested the Tao brush method as an attractive alternative to endometrial biopsy as it is just as minimally invasive but less uncomfortable^15^, ^20^ and more cost effective.^20^

The Tao brush cytology specimen is processed in a very similar manner to cervical cytology, which has been an established diagnostic method for several decades. Cervical cytology techniques continue to evolve with the incorporation of automation, artificial intelligence, and ancillary molecular tests (such as HPV) that have improved test performance.^23^, ^31^, ^32^, ^33^, ^34^ Likewise, endometrial cytology techniques are ripe for ancillary test development to enhance or replace manual cytomorphologic evaluation, but technology has yet to be developed or tested. For now, inter‐observer variability is a factor in the reading of endometrial cytology by pathologists and likely would require specialized training for those who would read this type of sample regularly. There remains little data on the cost of performance and interpretation of endometrial cytology specimens; however, one could extrapolate from cervical/pap literature that cytology specimens would be significantly less costly than histology with continuous improvements.^35^, ^36^

Meanwhile, the field continues to advance in the area of potential molecular testing (i.e., mutation, methylated DNA, FISH) which may serve to complement endometrial cytology in the future.^37^, ^38^ While the specificity of the Tao brush to detect EC and AH was not demonstrated to be as high as in some previous studies using similar modalities of testing,^16^, ^39^ a diagnostic test with lower specificity but comparable sensitivity may still be clinically beneficial if the sampling approach provides distinct advantages such as less discomfort and greater cost‐effectiveness. Tao endometrial brushing also has higher rates of successful insertion and tissue collection completion rate compared to traditional diagnostic methods of endometrial biopsy. In fact, in this study there were 14 patients who had inadequate endometrial biopsies; however, 12 of them had ample cells for cytological assessment. Alternately, endometrial brushing may be an opportunity to triage symptomatic patients to either traditional biopsy methods if cytology is inconclusive, surgery if clear that an EC is present, or no further intervention if cytology is clearly benign. This could spare a large proportion of women with AUB the greater discomfort associated with office endometrial biopsy compared to brushing.^15^, ^16^, ^29^

One of the strengths of this study was that each cytology specimen was reviewed by up to three pathologists with routine cytopathology experience but without extensive experience or training in endometrial cytology. This suggests that the learning curve for endometrial cytology may not be a limitation in the further development of endometrial cytology as a diagnostic test. The strength of interpretation was enhanced by the consensus of two pathologists; however, this is not routine in clinical practice for cervical cytology and may add expense due to additional pathologist time required if incorporated into clinical practice. To our knowledge, this is the first study assessing pathologist consensus among endometrial cytology specimens. Additionally, to our knowledge, this study is the largest thus far of women whose office endometrial biopsy and Tao endometrial brushing biospecimens have undergone direct comparison as well as the comparison to surgical specimen.

This present study was subjected to the areas of limitation. As previously mentioned, obtaining consensus was a study advantage, but the use of multiple pathologists may limit the generalizability to centers with solo practice or insufficient pathologists for multiple reviews. It is possible that multiple reviews could be eliminated with increased pathologist experience or automated processes. In our study, at least 45.7% (86 of 188 patients with times reported, and possibly up to 51.6% if we include the 11 with simultaneous times recorded) of patients had a biopsy performed first. This may have disrupted the endometrium enough to allow greater tissue collection by the Tao brush and could cause a false elevation in sensitivity. Using criteria A, the sensitivity of Tao brush for EC or AH was 100% (7/7),75% (6/8), and 100% (1/1) for those with endometrial biopsy first, Tao brush cytology first, and unknown timing, respectively (none of the patients with simultaneous times had EC or AH based on surgical specimen). However, these numbers are too small to draw any meaningful conclusions. Collection bias was also likely to present given that only patients who underwent clinically indicated surgery for diagnostic or therapeutic indications (AUB, PMB, abnormal ultrasound) were included in the cohort utilized to compare office collection techniques. Thus, final diagnoses demonstrated a higher proportion of EC and AH (nearly 16% combined) than would be expected in the setting of AUB and PMB. Caution should be utilized drawing conclusions in this area as this study was limited by the number of final EC and AH diagnoses.

In summary, given the promising sensitivity of the Tao brush in our study and the consistency among studies comparing this sampling method to endometrial biopsy, further study on its role as a triage and diagnostic method for the work‐up of AUB and PMB is warranted. With its potential for superior comfort, ease to learn, and cost‐effectiveness, endometrial cytology may offer distinct advantages above endometrial biopsy for the patient, for the provider, and for the health system as a whole. Given contemporary advances in molecular assays and automated cytology processing and analyses, revisiting endometrial cytology as a diagnostic tool is warranted.

## CONFLICT OF INTEREST

Dr. Bakkum has received Mayo Clinic CCSG grant and V Foundation funding. Dr. Bakkum and Dr. Wentzensen both have funding from the Intramural Research Program of the National Cancer Institute (grant number Z01CP010124‐21). Additional authors have no conflict of interest to disclose.

## AUTHOR CONTRIBUTIONS

All authors contributed to conceptualization, data curation, project administration, investigation, writing review, and editing.
